# Biogenic Cu/Ni nanotherapeutics from *Descurainia sophia* (L.) Webb ex Prantl seeds for the treatment of lung cancer

**DOI:** 10.1515/biol-2025-1160

**Published:** 2025-09-08

**Authors:** Xiao Zou, Jingsheng Chen, Jiaojiao Hu

**Affiliations:** Department of Internal Medicine, Wuhan University Hospital, Wuhan, Hubei, 430072, China; Department of Oncology, Ezhou Central Hospital, Ezhou, Hubei, 436000, China; Department of Critical Care Medicine, Gaoling Hospital Xi’an City Shaanxi Province, Xi’an, Shaanxi, 710200, China

**Keywords:** anti-lung carcinoma, cytotoxicity, antioxidant, bi-metallic NPs, copper, nickel

## Abstract

*Descurainia sophia* (L.) Webb ex Pran known as Flixweed is recognized as an ethnomedicinal plant in Chinese traditional medicine, offering numerous therapeutic benefits. Antioxidant chemicals found in this medicinal plant protect cellular integrity from various sources of damage and may help prevent cancer. In this study, we investigated copper/nickel nanoparticles (Cu/NiBMNPs@Flixweed) that were green-mediated following principles of green chemistry, utilizing the aqueous extract of *D. sophia* seeds for the treatment of lung carcinoma. The effectiveness of these Cu/Ni nanoparticles’ effectiveness was tested against three common human lung cancer cell lines. Methods such as X-ray diffraction (XRD), field emission-scanning electron microscopy (FE-SEM), Fourier transform-infrared spectroscopy, and energy dispersive X-ray were used to analyze the Cu/Ni nanoparticles produced through environmentally friendly methods. The XRD pattern revealed that the crystalline structure of the generated NPs is seen in the XRD pattern. According to the FE-SEM results, the nanoparticles had an average size of 68.52 nm and a semi-spherical shape. The IC_50_ values of Cu/NiBMNPs@Flixweed against HLC-1, LC-2/ad, and PC-14 cells were found to be 170, 98, and 57 μg/mL, respectively. The IC_50_ values of Cu/NiBMNPs@Flixweed against 2,2-diphenyl-1-picrylhydrazyl free radicals was 30 μg/mL. Recent research indicates that Cu/NiBMNPs@Flixweed may be a promising option to assist in the treatment various types of lung cancer.

## Graphical abstract

**Figure:**
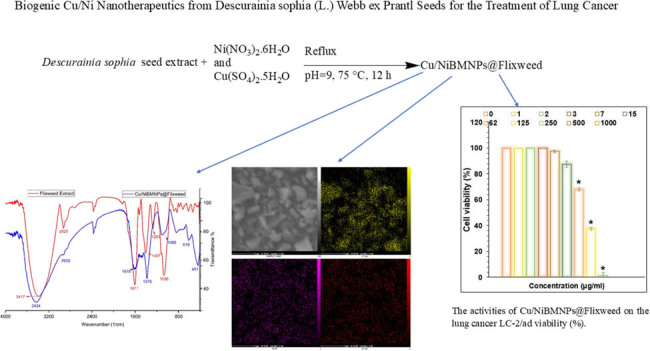

## 1 Introduction

Nanotechnology offers numerous benefits across various scientific disciplines. In this context, nanoparticles (NPs) are the fundamental components of nanotechnology [1–3]. Nanotechnology provides a wide range of advantages in different scientific fields. Within this framework, NPs serve as the essential building blocks of nanotechnology [4,5]. The ability to establish stable interactions with ligands, along with their diverse shapes and sizes, significant carrying capacity, and ease of binding to both hydrophobic and hydrophilic substances, make NPs ideal platforms for the controlled and targeted delivery of micro- and macromolecules in disease treatment [5–8]. NPs paired with therapeutic agents address challenges associated with traditional treatments. However, concerns regarding side effects and toxicity remain topics of discussion that should be carefully considered before their use in biological systems. Therefore, it is essential to understand the unique characteristics of therapeutic NPs and the methods used for their delivery [7–12]. Recent research has shown that NPs have beneficial therapeutic effects on various conditions, including cardiovascular issues, autoimmune disorders, infectious diseases, cancer, eye conditions, neurodegenerative diseases, and respiratory diseases [1,2,9–12]. Some research groups have investigated the anti-breast cancer and anti-gastric cancer effects of silver NPs using *Sambucus ebulus* rhizome and hull of *Pistacia vera* extracts and gold NPs green-mediated by *P. vera* extract, respectively [8,11]. Gaining insight into the properties of NPs and how they interact with biological systems will enable us to develop new approaches for treating, preventing, and diagnosing various diseases, especially those that are currently untreatable [1–4].

Bimetallic NPs are increasingly important in various fields due to their unique properties and enhanced functionalities compared to their monometallic counterparts. Composed of two different metals, bimetallic NPs can exhibit synergistic effects that improve their physical, chemical, and biological properties. These enhancements include increased catalytic activity, optical properties, and antimicrobial efficacy [13,14]. Bimetallic NPs have garnered attention for their antimicrobial properties, which outperform those of monometallic NPs. They have proven effective against a wide range of pathogens, making them promising candidates for medical applications, particularly in combating antibiotic-resistant bacteria. The dual metal composition allows for multiple mechanisms of action, decreasing the likelihood of microbial resistance [14,15]. In biomedicine, bimetallic NPs are utilized for drug delivery, imaging, and therapeutic agents. Their small size enables them to effectively penetrate biological barriers. Additionally, they can be engineered to release drugs in a controlled manner or enhance imaging contrast in diagnostic applications [15,16].

Because of their unique qualities and potential applications, nickel/copper bimetallic NPs, also known as Ni/Cu NPs, have recently garnered significant interest across various scientific fields. Ni/Cu NPs can be synthesized using different methods, such as chemical reduction and hydrothermal processes. The synthesis conditions, including the molar ratio of nickel to copper, can greatly impact the structural properties of the NPs [17–20]. One of the most noteworthy applications of Ni/Cu BMNPs is in catalysis. The bimetallic system often exhibits superior activity compared to its monometallic counterparts due to synergistic effects that enhance electron transfer rates and overall reaction efficiency [21]. Ni/Cu NPs have also been investigated for their antimicrobial properties. Studies have shown that these NPs can effectively inhibit the growth of various pathogenic microorganisms, surpassing individual nickel or copper NPs in terms of antibacterial efficacy. This is attributed to their unique physicochemical properties and mechanisms of action resulting from the bimetallic composition. They have demonstrated effectiveness against bacteria such as *Escherichia coli* and *Klebsiella pneumoniae*, making them potential candidates for medical and environmental applications [22,23].

There is an increasing demand for straightforward, cost-effective, and scalable methods to produce NPs. Utilizing plant extracts aligns with these requirements, as they are easy to cultivate in large quantities, renewable, and environmentally sustainable [24]. In the process of synthesizing metallic NPs, phytochemicals serve dual functions: they act as both reducing agents and stabilizers for the NPs [24,25]. Bimetallic NPs synthesized using plant extracts exhibit enhanced stability and diversity in their shapes and sizes. Consequently, the presence of reducing agents in these extracts such as flavonoids, terpenoids, and phenolic acids is crucial for the successful synthesis of bimetallic NPs [26].

The Brassicaceae family, commonly known as the mustard family, consists of approximately 338 genera and around 3,700 species of flowering plants. This family is well known for its edible members, which are widely consumed as vegetables worldwide and are valued for their high content of bioactive phytochemicals. Notable vegetables from this family include Brussels sprouts, cabbage, broccoli, and kale, among others [27]. *Descurainia sophia* holds significant importance in traditional medicines globally, where it is highly regarded among practitioners of folk medicine for its extensive use in various remedies [28,29]. *D. sophia* is prevalent in northeastern China and has a long history of use in traditional folk medicine. This plant is notorious for being a broadleaf weed that infests winter wheat fields in the region. Notably, it has developed resistance to the acetolactate synthase-inhibiting herbicide tribenuron-methyl, posing a significant challenge for agricultural management in these areas [30]. The decoction made from the aerial parts of *D. sophia* is used to treat throat ailments and acts as an antipyretic in cases of measles and smallpox. This traditional application highlights the plant’s importance in herbal medicine, particularly in addressing fever and respiratory issues associated with these diseases [31]. There is substantial evidence highlighting the therapeutic properties of *D. sophia* for a variety of health issues, including asthma, cough, urinary tract disorders, pain, edema, cardiac conditions, constipation, fever, itching, intestinal parasites, and internal bleeding. Additionally, the seeds of *D. sophia* are utilized to address various pathological conditions such as gastrointestinal issues, inflammation, and cardiovascular diseases. The plant is also suggested as a treatment for vitamin C deficiency. *D. sophia* has been observed to exhibit significant cytotoxic effects against a variety of human cancer cell lines, indicating its potential as an anticancer agent. Studies have documented its efficacy in targeting cancer cells from the lung, liver, colon, prostate, ovary, skin, and stomach. Also, the *D. sophia* seeds ethanolic extract has shown the ability to suppress cancer cell growth and induce apoptosis, particularly in lung cancer cells, by regulating genes associated with cell growth signaling pathways. This suggests that *D. sophia* may serve as a promising candidate for the development of anticancer therapies [32–35]. *D. sophia* seed extract is known for its potent cytotoxic effects against lung cancer cells [36,37]. The diverse medicinal uses of *D. sophia* are attributed to the presence of various secondary metabolites, including phenolics, cardiac glycosides, flavonoids, and sulfur glycosides [31,36,38]. These compounds play a crucial role in the plant’s therapeutic effects, contributing to its efficacy in treating a range of health disorders. The phytochemical profile of *D. sophia* supports its traditional applications in medicine, highlighting its potential as a source of bioactive substances for future pharmacological development [36,38]. So far, silver and gold NPs have been synthesized using *D. sophia* extract. The gold NPs showed acceptable ability to treat ovalbumin-induced asthma [39]. The silver NPs were found to be active against lung cancer cell lines [40]. The NPs also exhibited antimicrobial and antifungal activity [41].

In recent years, the synthesis of metallic NPs using plant extracts has gained desirable attentions. This is primarily due to the green, eco-friendly, and cost-effective nature of this approach compared to conventional physical and chemical methods. Bimetallic NPs have shown promising potential for lung cancer treatment due to their unique properties and advantages. In this study, we focused on the green synthesis of bimetallic NPs of copper and nickel using the extract of the seeds of *D. sophia* as a reducing and stabilizing agent. This method provides a green, eco-friendly, and cost-effective alternative, avoiding toxic chemicals typically used in NP synthesis, and the resulting biocompatible NPs are safer for biomedical applications. By synthesizing Ni/Cu NPs using *D. sophia* seed extract, we leverage the plant’s natural anticancer compounds and the unique properties of metal NPs to create a green, stable, and effective therapeutic agent with enhanced cytotoxicity against lung cancer cells. The physio-chemical characteristics of the NPs were evaluated using analytical methods. Furthermore, the application of NPs in preventing lung cancer, cytotoxicity, and antioxidant properties was assessed. However, a major gap in the present study lies in the clinical evaluation of these specific NPs against lung cancer. Lung cancer is a complex disease, and the efficacy, targeting ability, and safety of these specific NPs for lung cancer treatment would require rigorous testing.

## 2 Experimental

### 2.1 Plant and chemical materials

The seed of *D. Sophia* was purchased from a medicinal plant store in Wuhan, China. The plant was identified by a botanist in the biology section of Wuhan University with a voucher specimen of WU1054. The plant seeds were washed and dried in a dark place at room temperature.

All chemicals for the present study were purchased from Sigma Aldrich with analytical grade.

### 2.2 Chemical characterization

The Fourier transform-infrared spectroscopy (FT-IR) spectra were recorded using a Shimadzu 8400 spectrophotometer with KBr disc ranging from 400 to 4,000 cm^−1^. The X-ray diffraction (XRD) diagram was obtained on the 2*θ* scale using a STOE PW2773.00 device with Cu Kα radiation at 45 kV and 40 mA. The field emission-scanning electron microscopy (FE-SEM) images and energy dispersive X-ray (EDX) data were obtained using a MIRA3TESCAN instrument.

### 2.3 Synthesis of Cu/NiBMNPs@Flixweed

The synthesis was carried out according to a previous study with some modification [42]. First, 5 g of flixweed seeds were boiled in deionized water (200 mL) for 15 min. After reaching ambient temperature, the extract was filtered. To synthesize Cu/NiBMNPs@Flixweed, 15 mL of the extract was mixed with Ni(NO_3_)_2_·6H_2_O and Cu(SO_4_)·5H_2_O in a 1:1 ratio at a concentration of 0.05 M. The pH was adjusted to 9. The reaction mixture was refluxed at 75°C for 12 h. The residue was then centrifuged at 10,000 RPM for 10 min. The NPs were centrifuged with four water washes to eliminate unreacted components. Finally, dried for 6 h at 55°C in an oven before processing for characterization and biological tests. The synthesis of NPs was repeated five times.

### 2.4 Anti-lung cancer efficacy of Cu/NiBMNPs@Flixweed

The cytotoxic effects of Cu/NiBMNPs@Flixweed was evaluated on the PC-14 (origin source: human lung, accession number: CVCL_1640), LC-2/ad (origin source: human lung, accession number: CVCL_1373), and HLC-1 (origin source: human lung, accession number: CVCL_5529) purchased from Shenzhn Bike Biotechnology Company, China. Lung carcinoma cells were measured using the 3-[4,5-dimethylthiazol-2-yl]-2,5 diphenyl tetrazolium bromide (MTT) assay. Cu/NiBMNPs@Flixweed were applied to the cells for 24 h at various dosages from 1 to 1,000 µg/mL. To each well 100 µL of 0.5 mg/mL MTT solution was added to replace the medium for cytotoxicity assessment. After 4 h of dark incubation at 37°C, the medium was removed and 0.1 mL of dimethyl sulfoxide was added to each well and mixed well for an additional 10 min. A microplate reader (Bio-Rad, CA, USA) was used to measure the absorbance at 570 nm. Cell viability was calculated using the following equation [43]:

(1) \[{\mathrm{Cell\; viability}}(\left \% )=\frac{{\mathrm{Sample\; Absorption}}}{{\mathrm{Control\; Absorption}}}{\mathrm{\times }}100.]\]

The program GraphPad Prism version 9 was used to calculate the half-maximal inhibitory concentration (IC_50_) values. Additionally, a digital camera-equipped phase-contrast inverted microscope (Olympus, Japan) was utilized to observe the cellular morphology of both untreated and treated cells.

### 2.5 Antioxidant efficacy of Cu/NiBMNPs@Flixweed

The antioxidant activity of Cu/NiBMNPs@Flixweed was examined using the 2,2-diphenyl-1-picrylhydrazyl (DPPH) free radical test, based on techniques from a previous study [43]. When a material capable of donating hydrogen atoms was added to the alcoholic DPPH solution, the DPPH decreased and turned pale yellow instead of purple. Two milliliters of a freshly made methanolic solution (0.1 Mm) of DPPH was mixed with 2 mL of Cu/NiBMNPs@Flixweed solution (1–1,000 µg/mL). The test tubes were then placed in the dark for half an hour and absorbance was measured at 517 nm after incubation. Butylated hydroxytoluene (BHT) at concentrations ranging from 1 to 1,000 µg/mL was used as a reference or control. A reaction mixture showing lower absorbance indicates greater free radical scavenging activity [43]. Using BHT as a reference, equation (2) was used to calculate the antioxidant capabilities of the samples based on their DPPH radical scavenging abilities:

(2) \[{\mathrm{SCV}}(\left \% )=\frac{({\mathrm{Ac}}-{\mathrm{As}})}{{\mathrm{Ac}}}{\mathrm{\times }}100,]\]

where SCV represents the DPPH radical scavenging effect, and AS and AC denote the absorbance of the sample and control, respectively.

### 2.6 Statistical analysis

GraphPad Prism Version 9.0 was utilized for the graphical analysis of the data, presenting the results as mean ± standard deviation. The statistical significance was evaluated using one-way ANOVA followed by the Tukey–Kramer Multiple Comparison Test, with a *p*-value of ≤0.01 denoted by an asterisk.

## 3 Results and discussion

**Scheme 1:**
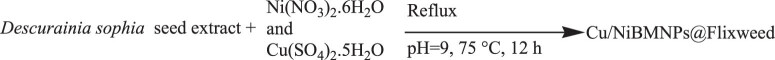
Synthesis procedure of Cu/NiBMNPs@Flixweed.

Scheme 1 presents the chemical process of the synthesis for bi-metallic NPs of copper and nickel using the aqueous extract of *D. sophia* (Cu/NiBMNPs@Flixweed). The reaction was optimized using different factors such as pH values of 7, 8, 9, and 10; temperatures of 45, 60, 75, and 90°C; and varying concentrations of metallic salts (0.03, 0.04, 0.05, 0.06, 0.07 M). A comparison of the yield percentages revealed the optimal conditions for the synthesis of Cu/NiBMNPs@Flixweed to be pH 9, temperature 75°C, and metallic salt concentration 0.05 M.

**Figure 1:**
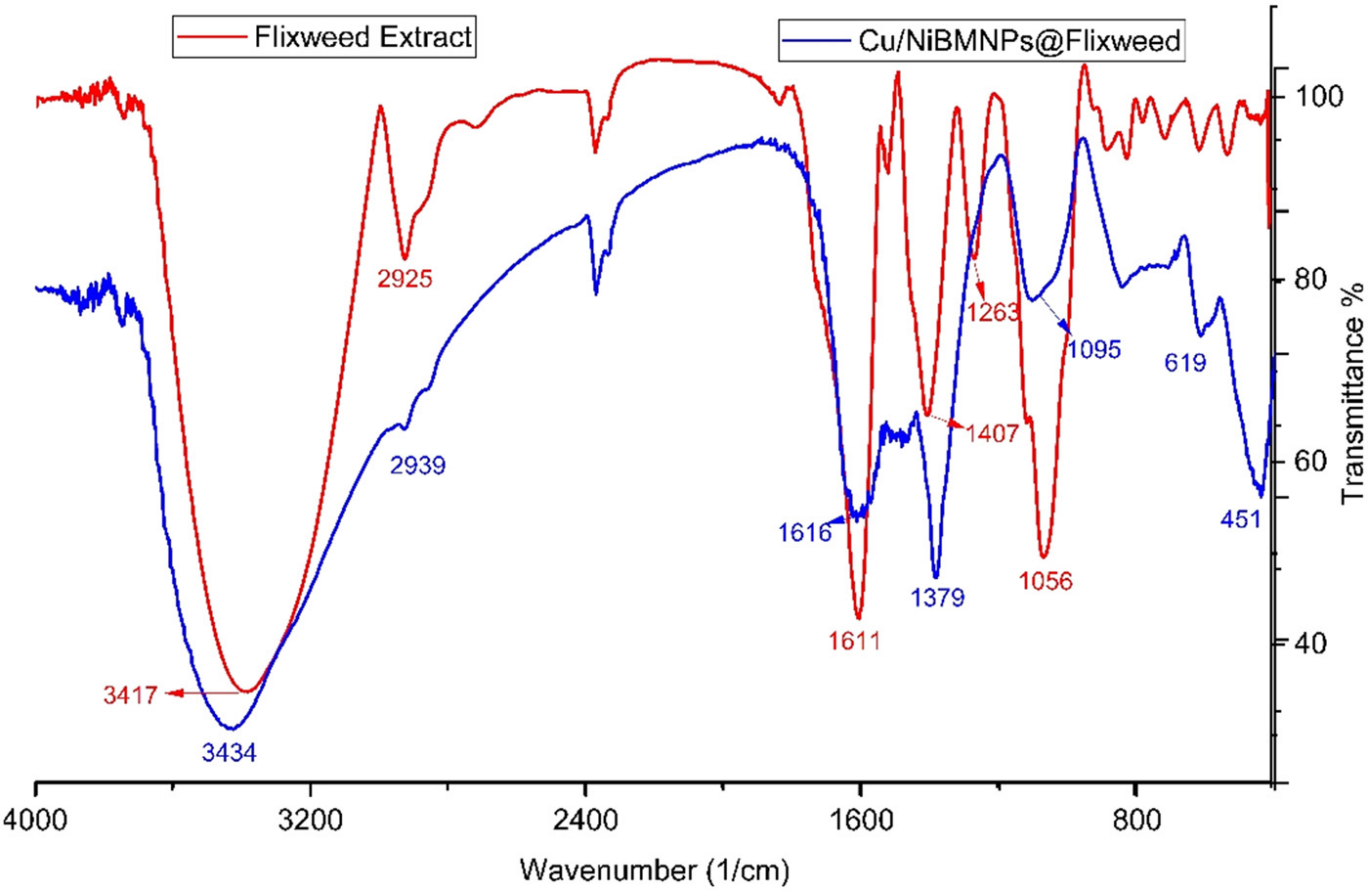
Cu/NiBMNPs@Flixweed FT-IR spectrum.

FT-IR is an effective technique for the characterization of metallic NPs. It offers important details on the functional groups and chemical makeup of the NPs, which are essential for understanding their characteristics and uses. The technique can be used to monitor any structural changes in metallic NPs upon exposure to various conditions (e.g., temperature, pH, or biological environments). Changes in peak positions or intensities in the FT-IR spectrum can indicate modifications in the chemical structure or bonding environment around the NPs [44,45]. Figure 1 presents the FT-IR spectra of Cu/NiBMNPs@Flixweed and the plant extract. The obtained results authenticate the synthesis of copper/nickel bi-metallic NPs with a linkage of the organic compounds of Flixweed extract to the surface of NPs. The bands at 451 and 619 cm^−1^ correlate with Cu–O and Ni–O bonds. These results are in agreement with previous reports on the green synthesis of Cu/Ni BMNPs [46,47]. Other bands at various wavenumbers reveal the coating of the NPs surface by the secondary metabolites of Flixweed extract, with evidence provided by the bands related to different organic functional groups. The peaks for functional groups of O–H (3,434 cm^−1^), C–H (2,939 cm^−1^), C═C and C═O (1,379–1,616 cm^−1^), and C–O (1,095 cm^−1^) are reported in the FT-IR spectrum of Cu/NiBMNPs@Flixweed. These signals closely match the bands of functional groups which were recorded for the plant extract, with slight shifts in peak positions. The results show bands at 3,417, 2,925, 1,407–1,611, 1,263, and 1,056 cm^−1^ for vibrational bands of O–H, C–H, C═C, C═O, and C–O.

**Figure 2:**
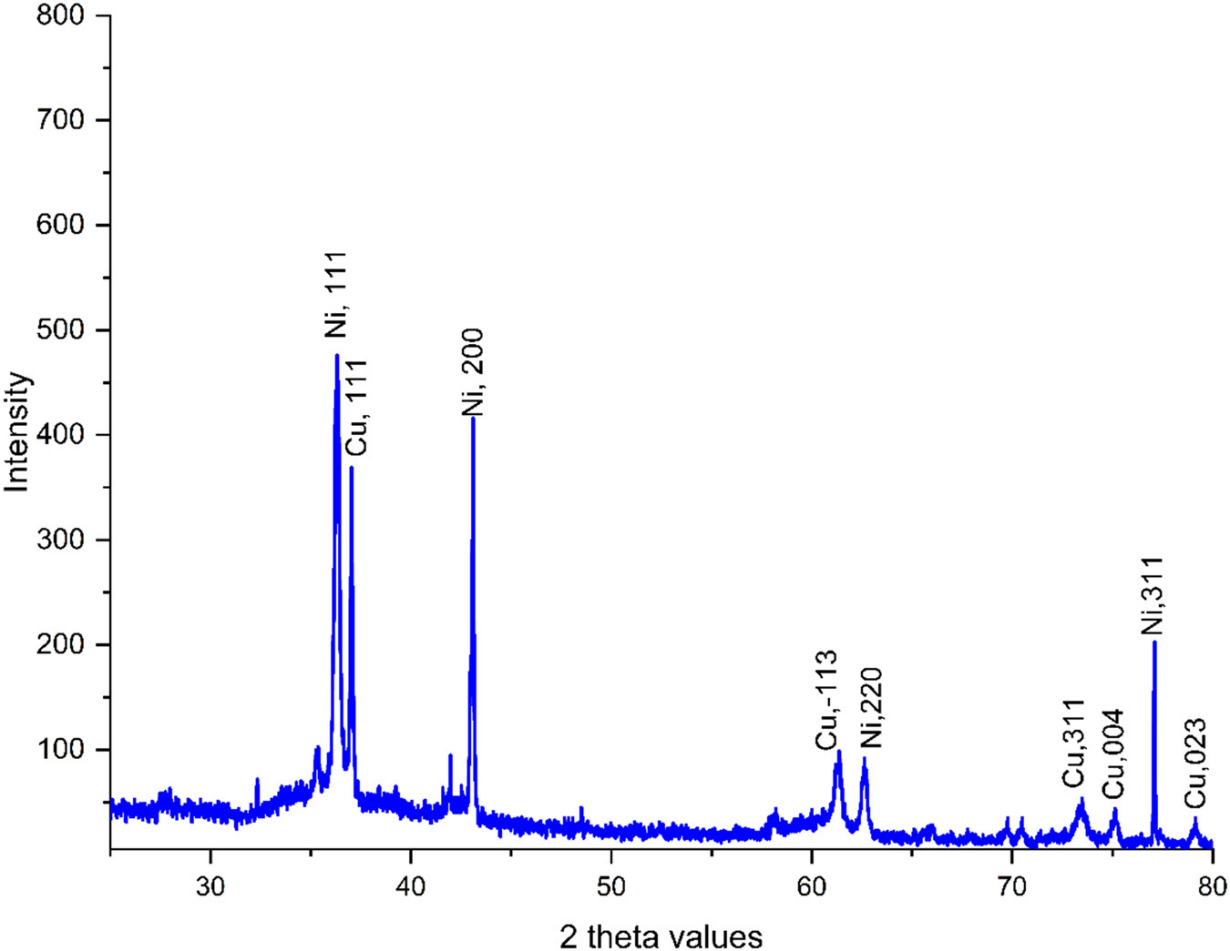
Cu/NiBMNPs@Flixweed XRD diagram.

XRD is a widely used technique for the characterization of NPs, providing essential information about their structural properties. XRD is primarily employed to determine the crystalline structure of NPs. The technique helps to identify the phases present in a sample by comparing the observed diffraction peaks with standard reference patterns from databases. This phase identification is crucial for understanding the material properties and potential applications of the NPs [48]. Figure 2 shows the XRD diagram of Cu/NiBMNPs@Flixweed. According to the results, the NPs were materialized in the crystalline structure. The signals at different positions match the standard data of copper oxide (CuO) and nickel oxide (NiO). However, due to the formation of bimetallic NPs, slight differences in the positions of signals are perceptible. The signals at 37.03 (111), 48.49 (−202), 61.03 (−113), 73.34 (311), 74.97 (004), and 79.07 (023) match the PDF card No. 04-012-7238 for CuO and the signals at 36.28 (111), 43.11 (200), 62.76 (220), and 77.02 (311) are compatible with the standard data of JCPDS No. 1313-991 for NiO. The obtained data are similar to previous reports for Cu/Ni NPs [46,47,49].

**Figure 3:**
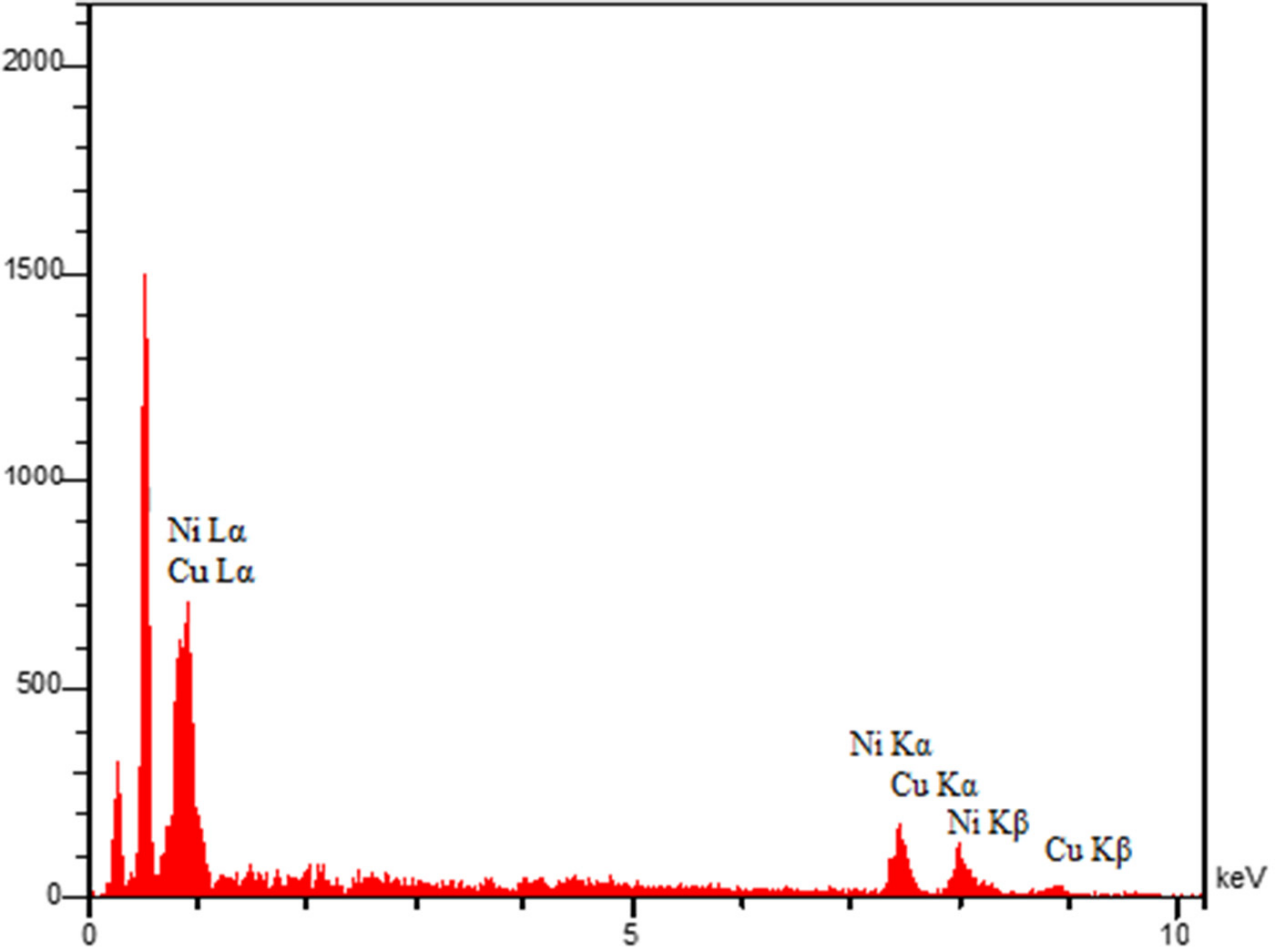
Cu/NiBMNPs@Flixweed EDX diagram.

**Figure 4:**
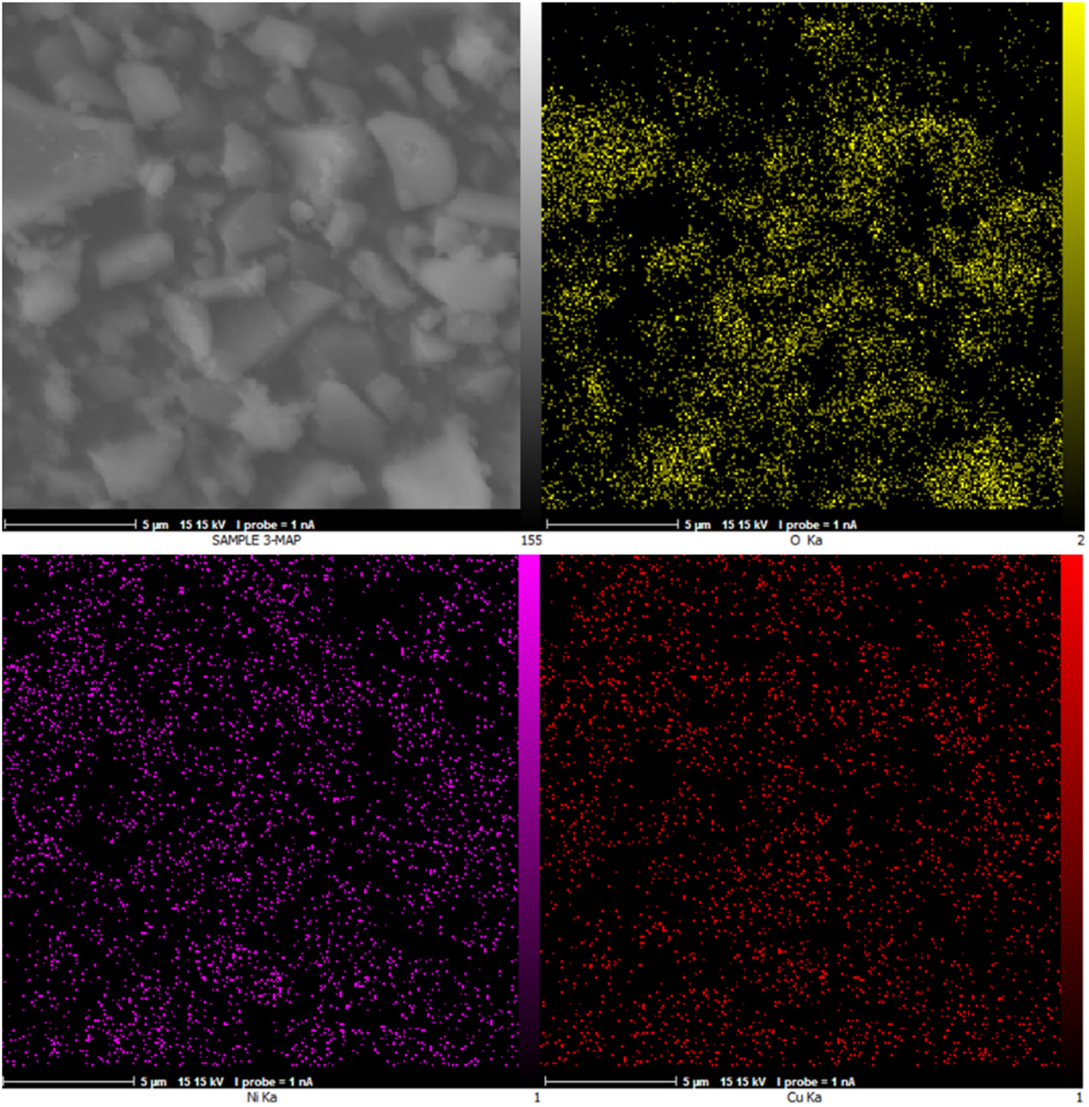
Cu/NiBMNPs@Flixweed EDX mapping.

EDX spectroscopy is an essential technique for the characterization of NPs, providing critical insights into their elemental composition and distribution. Energy dispersive X-ray spectroscopy allows for the determination of the elemental composition of NPs by analyzing the sample’s characteristic X-rays emitted when excited by an electron beam. Researchers can identify the types and relative abundances of elements present in the NPs, crucial for confirming the successful synthesis and composition of metallic NPs [50]. The EDX analysis results of Cu/NiBMNPs@Flixweed are shown in Figure 3, indicating signals for copper (around 0.9 keV for Cu Lα, 7.5 keV for Cu Kα, and 9 keV for Cu Kβ) and nickel (around 0.8 keV for Ni Lα, 7.6 keV for Ni Kα, and 8.2 keV for Ni Kβ). Furthermore, signals for carbon or oxygen from organic compounds demonstrate the importance of flixweed secondary metabolites as the reducing and capping agent for the synthesis of Cu/NiBMNPs@Flixweed. Compared to previous reports on the green synthesis of copper, nickel, and Cu/Ni, the results confirm the successful synthesis of Cu/Ni NPs [47,51,52]. EDX mapping is a powerful technique used in the characterization of NPs, allowing for the identification of the elemental composition of NPs crucial in understanding their properties and behavior. It also provides information on the spatial distribution of elements within the NPs [53]. The EDX diagram of Cu/NiBMNPs@Flixweed is exhibited in Figure 4 showing the uniform distribution of copper, nickel, and oxygen elements in NPs.

**Figure 5:**
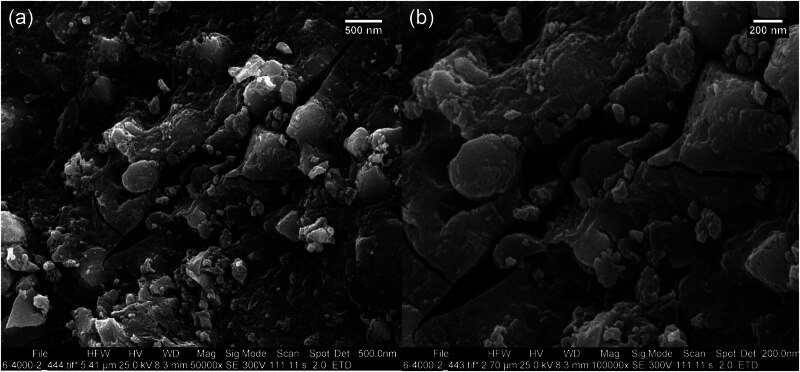
Cu/NiBMNPs@Flixweed FE-SEM images. (a) 500 nm; (b) 200 nm.

FE-SEM is a tool used to characterize green-formulated metallic NPs. It provides high-resolution imaging that reveals important information about the morphology, size, and distribution of these NPs. FE-SEM allows for a detailed visualization of the shape and surface characteristics of green-synthesized metallic NPs, which is crucial for understanding how their morphology affects their properties and potential applications. For example, spherical, rod-shaped, or irregularly shaped NPs can exhibit different behaviors in biological or catalytic contexts [54,55]. The FE-SEM images of Cu/NiBMNPs@Flixweed are shown in Figure 5, illustrating a semi-spherical morphology for Cu/NiBMNPs@Flixweed with an average size of 68.52 nm. The NPs display a tendency to aggregate, similar to other metallic NPs reported previously [56–59]. Aggregation typically occurs when the stabilizing agents such as organic compounds from the plant extract that prevent NPs from clumping are disrupted [60].

Recent gas chromatography and gas chromatography–mass spectrometry analysis of the volatile components in the aerial parts of *D. sophia* revealed that neoisomenthyl acetate, menthol, and cis-β-ocimene are the main components of the volatile oil [36]. Studies have shown that the seeds of *D. sophia* can inhibit the growth of various types of cancer cells. Specifically, the viability of SF268, NCI-H460, and SGC-7901 cells was significantly reduced by n-butanol extract of the seeds [61,62]. Previous research has found that artabotryside A, a flavanol glycoside isolated from *D. sophia* seeds, halted the growth of U87 glioblastoma cells by inducing G2/M phase arrest and caspase-3-dependent cell death [36]. In A549 lung cancer cells, Kim et al. demonstrated that the ethanol extract from *D. sophia* seeds showed dose-dependent efficacy [36]. BGC-823 and MDA-MB-435 cells were both found to be cytotoxically affected by some of the antioxidant chemicals present in *D. sophia* seeds [63]. The hydrolyzed derivatives of glucosinolates, specifically isothiocyanates, have exhibited antioxidant, antibacterial, anticancer, and chemoprotective properties. It has been consistently shown that consuming plants high in isothiocyanates can reduce the risk of developing cancer [64]. By aiding in detoxification, which reduces the activation of pro-carcinogens and enhances the removal of carcinogens, isothiocyanates may offer cancer prevention. A diet rich in cruciferous vegetables has also been shown to increase detoxifying enzymes. Additionally, isothiocyanates may reduce tumor development by blocking the CYP-dependent activation of pre-carcinogens promoting, or inhibiting the proliferation of cancer cells [65].

**Figure 6:**
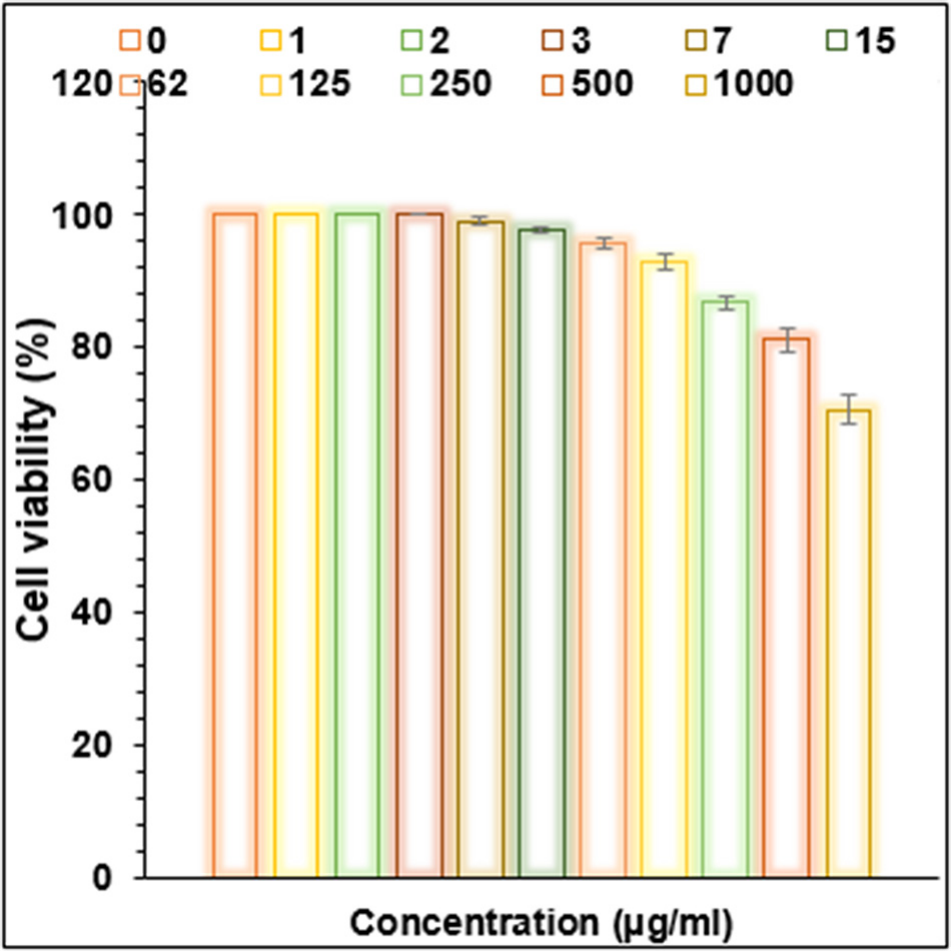
Activities of Cu/NiBMNPs@Flixweed on the normal cell viability (%).

**Figure 7:**
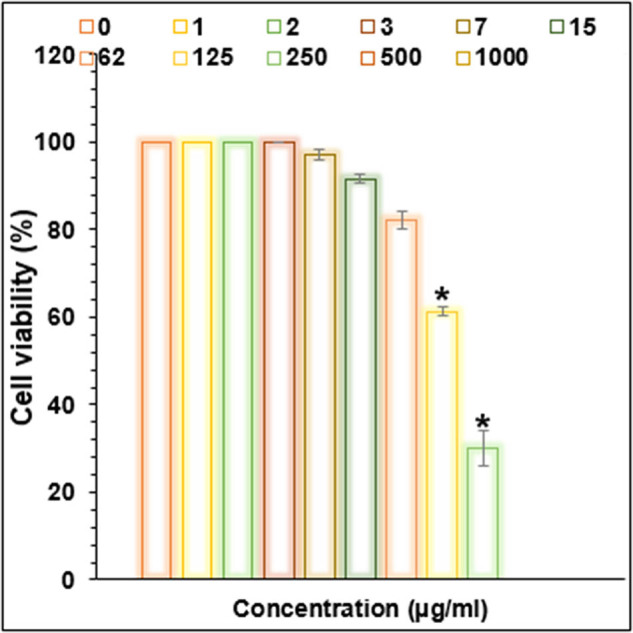
Activities of Cu/NiBMNPs@Flixweed on the lung cancer HLC-1 viability (%).

**Figure 8:**
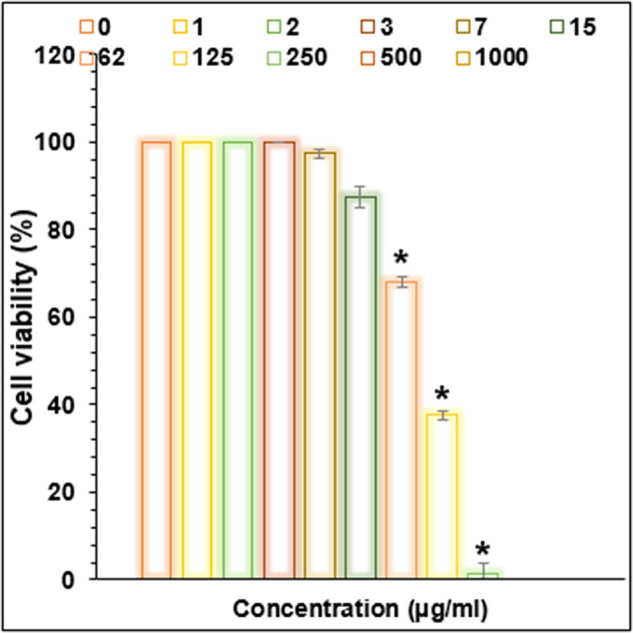
Activities of Cu/NiBMNPs@Flixweed on the lung cancer LC-2/ad viability (%).

**Figure 9:**
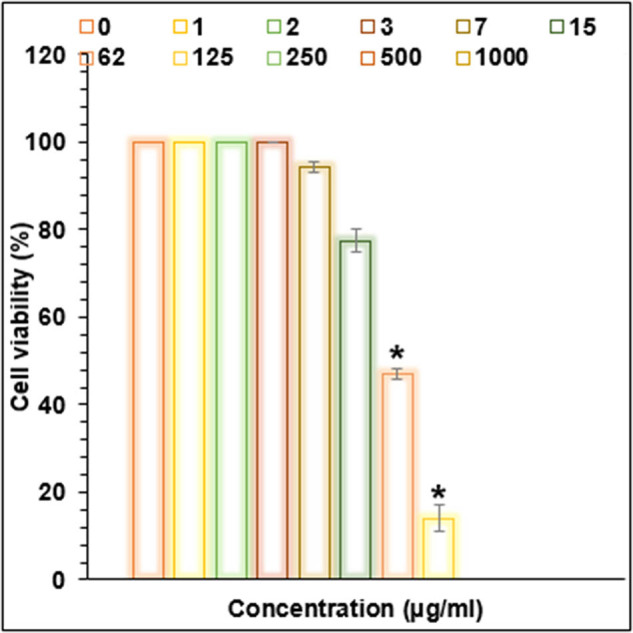
Activities of Cu/NiBMNPs@Flixweed on the lung cancer PC-14 viability (%).

Cu/NiBMNPs@Flixweed significantly decreased the viability of cancer cells in this study (Figures 6–9). The viability of tumor cells decreases from 100 to 0% as the concentration of Cu/NiBMNPs@Flixweed increased from 0 to 1,000 μg/mL. PC-14, LC-2/ad, and HLC-1 had respective IC_50_ values of 57, 98, and 170 μg/mL, respectively. Cu/NiBMNPs@Flixweed exhibit greater cytotoxicity toward cancer cell lines due to its superior stability and cellular absorption. Since Cu/NiBMNPs@Flixweed are smaller and have a larger surface area, they may enter cells more readily by endocytosis and circumvent the p-glycoprotein efflux mechanism [66]. Biosynthesized NPs have shown great promise in the fight against cancer cell lines, according to earlier studies [67,68]. Recent research by AshaRani et al. and Nagajyothi et al. suggests that environmentally friendly NPs may effectively stop the growth of MCF-7 cells, A549 cells, and human glioblastoma cells. The precise mechanism by which cancer cells function is yet unclear [69,70]. According to Xu et al. a hydrogel containing NPs damaged DNA and raised the generation of reactive oxygen species (ROS) in cancer cells, which eventually led to cell death. Recent research suggests that NPs may have harmful effects on healthy cells. However, the level of cytotoxicity is influenced by variables such as particle size and concentration [71]. Larger particles are generally less dangerous than tiny ones, and higher concentrations are more likely to have adverse consequences. It is crucial to remember that dosage determines how harmful NPs are, and the quantities employed in research may not accurately reflect exposure levels seen in daily life. Additionally, the results may change based on the kind of normal cells examined since various cell types may respond to NPs differently [72]. So far, a few studies have focused on the anti-lung cancer activity of bi-metallic NPs. These materials show promising anti-lung cancer activities primarily through inducing cytotoxicity and affecting cancer cell survival pathways. They have been found to cause dose-dependent cytotoxic effects on non-small cell lung cancer cell lines reducing cell viability significantly. They induce oxidative stress and programmed cell death selectively in cancer cells, sparing healthy cells due to the enhanced permeability and retention effect that allows NPs to accumulate in tumor tissues. They also demonstrate antioxidant properties by reducing ROS and interfering with key cancer survival signaling pathways [73–76].

**Figure 10:**
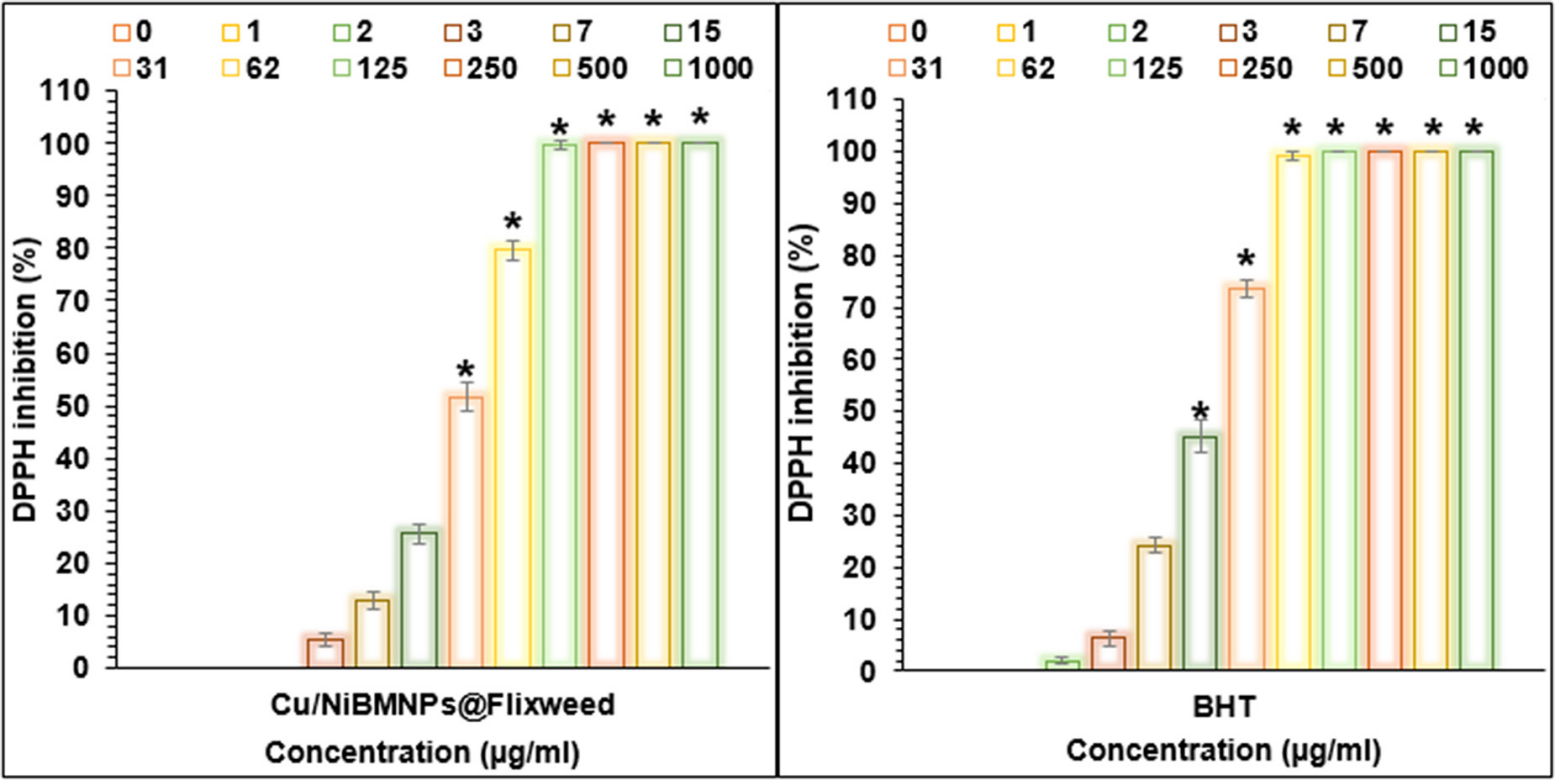
Antioxidant activities of Cu/NiBMNPs@Flixweed on DPPH inhibition (%).

A DPPH experiment was conducted to evaluate the antioxidant potential of BHT and NPs. Antioxidant properties varied significantly and increased in a dose-dependent manner (Figure 10). At the highest doses of 250, 500, and 1,000 μg/mL, the reported value of Cu/NiBMNPs@Flixweed increased from 5.3% at the lowest concentration of 3 μg/mL to 100%. The antioxidant results are presented using IC_50_ values (Figure 10). The IC_50_ value of Cu/NiBMNPs@Flixweed is 30 μg/mL. A lower IC_50_ indicates greater antioxidant capacity. Cu/NiBMNPs@Flixweed’s surface contains a variety of functional groups that may enhance its antioxidant properties. Based on these findings, Cu/NiBMNPs@Flixweed could potentially be used as a substitute antioxidant for treating illnesses caused by free radicals. The strong antioxidant properties of NPs have been demonstrated in several studies [38,60,61]. When free radicals obtain hydrogen from the proteins, phenols, alkaloids, and other compounds present in *D. Sophia*, stable phenoxyl radicals are generated [56–60].

## 4 Conclusion

Copper/nickel bimetallic NPs were prepared using a green chemistry approach with flixweed seed extract. The NPs were characterized using SEM, XRD, FT-IR, and EDX. Results indicated an interaction between the plant extract and metallic ions during synthesis, resulting in reduced NPs with a crystal structure, semi-spherical morphology, and an average size of 50.55 nm. Tests on Cu/NiBMNPs@Flixweed showed antioxidant efficacy against free radicals ranging from 5.3 to 100%. The effectiveness against lung cells decreased with increasing NP concentration, demonstrating the anti-lung cancer properties of Cu/NiBMNPs@Flixweed. According to the findings of the *in vitro* study, several concentrations of Cu/NiBMNPs@Flixweed had notable anti-lung cancer effects with low toxicity against normal cells suggesting potential in cancer treatment.

The anti-lung cancer and antioxidant properties of Cu/NiBMNPs@Flixweed suggest they be utilized as a supplementary treatment globally. However, thorough safety assessments are necessary due to the unique properties of bimetallic NPs. Understanding their absorption, distribution, metabolism, and excretion in living system (pharmacokinetics) and their effects on the body (pharmacodynamics) is crucial for clinical translation. This research highlights a promising path for future studies combining the benefits of flixweed with anti-cancer activity of Cu/Ni NPs.
